# Book Review: A Handbook for Student Engagement in Higher Education: Theory Into Practice

**DOI:** 10.3389/fpsyg.2021.717026

**Published:** 2021-06-29

**Authors:** Bing Zhang, Zhongmin Yu

**Affiliations:** ^1^Institute of Physical Education, Huanggang Normal University, Huanggang, China; ^2^Huanggang Institute of Education Science, Huangzhou, China

**Keywords:** student engagement, higher education, theory into practice, learning, teaching

Tom Lowe and Yassein El Hakim (New York, NY: Routledge), 2020, xix+303 pages, ISBN: 978-0-367-08543-8

Student engagement has been a buzzword for many years in education and has been uncritically regarded as an academic orthodoxy. However, reconciling theory and practice in student engagement in higher education is a long-felt gap, which is perspicaciously filled by *A Handbook for Student Engagement in Higher Education: Theory into Practice*, eloquently edited by Tom Lowe and Yassein El Hakim. Student engagement plays a significant role in students' success and motivation, highly affected by teacher-student interpersonal variables. which is affected.

This voluminous handbook comprises 23 chapters, thematically organized into four parts. Part I, containing six chapters, thoroughly introduces engagement in educational developments (SEEDs), looking at it from different perspectives. The editors open up this compendium by presenting a panoramic overview of the germane issues in SEEDs, sketching the historical change, and calling for a more critical perspective. The authors, in Chapter 2, argue that roles, position, and power of students need to be taken into account to build and sustain relationships with students, professors, and staff for SEEDs. The essence of Chapter 3 is “How do we ensure that non-traditional students are considered when planning student engagement strategy?” (p. 47); the author cogently argues “we need to ask them, work with them and be inspired by them to facilitate positive engagement for all.” (p. 47). Chapter 4 recapitulates the theories and principles underlying SEEDs, accentuating that to ascertain that learner involvement initiatives are prosperous, “participants in them need to perceive them as if they are *authentically inclusive, transparent, trusting and empowering*” (Italics are original. p. 61). Chapter 5 highlights the changing nature and significance of learner manifestation by focusing on individual and collective student engagement. Chapter 6 critically looks at SEEDs from an assessment perspective, endorsing the opportunities for learners to assess their learning and teaching.

Part II consists of five chapters. Conceptualizing a fruitful model for SEEDs, Chapter 7 encourages us to focus on student involvement via classroom-oriented pedagogical partnership. Informed by the 4M (Micro, Meso, Macro, and Mega) framework, Chapter 8 examines how these four levels are instantiated in evaluating student-staff partnership as well as institutional changes and educational advancements. Chapter 9 presents the outcome of implementing a pilot work-integrated learning program, motivating learners to utilize their present non-science work as an impetus for their reflection. Chapter 10 elaborates on the features of Meaningful Student Involvement (MSI), problematizes its challenges, and concludes that “Rather than alienating students from the process of educational transformation, we should seek to do nothing less than enliven any level of interest students may have in the first place” (p. 146). Chapter 11 contends that learner involvement as a heuristic is truly problematic “on issues of both student identity (who?) and activity (doing what?)” (p. 149), advocating that learners are eventually the agents of discussions of involvement.

Part III, encompassing eight chapters, pertains to the practical models of student engagement. Prioritizing championing learners' voices, learners as partners, and learners as collaborators, Chapter 12 supports the view that working individually is cumbersome, and we are advised to work in collaboration with other responsible bodies in HE. Like Chapter 12, Chapter 13 scrutinizes how student voices can transform their learning experiences and can culminate in more student engagement. Chapter 14 discusses that student engagement can be maximized “by supporting and normalizing grassroots partnership, where there are mechanisms in place to support students and staff who want to work in partnership in their own contexts and not necessarily be attached to an initiative” (p. 183). The other chapters in this section aim to unpack how students can be digitally empowered in technology-enhanced learning (Chapter 15) and how they can be empowered to boost their education (Chapter 16) through partnership initiatives. Enlightened Peer-Assisted Student Success initiatives, Chapter 17 reports the findings of a qualitative study, aiming to find out how these individuals become Success Coaches, how they become engaged in learning, and how they can promote agency. Besides, Chapter 18 represents that learner partnership schemes that can act as digital change agents are effective in student engagement. Similarly, Chapter 19 argues that scholarship can be an effective impetus to boost student engagement.

Part IV, comprising four chapters, is concerned with the future of learner engagement. Taking a personal stance, the author in Chapter 20 argues that the future of learner involvement needs to realize the multidimensional nature of student success. Chapter 21 cogently recommends “the need to find space to be critically reflective; not to forget the distinction between *students engaging* and *engaging students*; to actively oppose appropriation of student engagement” (p. 263). The penultimate chapter suggests that issues such as cooperative partnerships, technology-enhanced initiatives, and student representativeness should be emphasized. Chapter 23 highlights the role of leadership, collaboration, equity, and empowerment in student engagement.

The editors of this handbook need to be commended for this meritorious compendium on different grounds. First, the handbook encompasses diverse theoretically-driven and empirically-grounded investigations which pave the way for future research on student engagement. Secondly, the handbook highlights the role of different student engagement initiatives which can act as springboards for teachers and researchers. Foregrounding the role of students' voices in their engagement in different chapters is another merit of this collection. However, what remains untouched in this volume is the role of student engagement in language classes (Hiver et al., 2021). Besides, more can be added about the other dimensions of student engagement, namely academic, affective, behavioral, cognitive, and social.

Taken it together, this contemporary handbook on student engagement is a treasure trove for teachers, students, teacher educators, curriculum developers, policymakers, and HE staff who are willing to maximize student engagement.

## Author Contributions

BZ was in charge of writing. Both authors make contribution to the research design.

## Conflict of Interest

The authors declare that the research was conducted in the absence of any commercial or financial relationships that could be construed as a potential conflict of interest.
